# Low Shrinkage Transparent UV-Cured 3D Printing Hard Silicone Resins

**DOI:** 10.3390/polym17081123

**Published:** 2025-04-21

**Authors:** Haibo Wu, Qili Shen, Zhu Liu, Xiantai Zhou, Yanxiong Fang, Hongping Xiang, Xiaoxuan Liu

**Affiliations:** 1 School of Chemical Engineering and Light Industry, Guangdong University of Technology, Guangzhou 510006, China; wu.haibo08@163.com (H.W.); qlshen1216@163.com (Q.S.); xianghongping@gdut.edu.cn (H.X.); p-xxliu@gdut.edu.cn (X.L.); 2 School of Dayawan Chemical and New Materials, School of Chemistry and Materials Engineering, Huizhou University, Huizhou 516007, China; 3 Huizhou Research Institute of Sun Yat-sen University, Sun Yat-sen University, Huizhou 516084, China; 4 Guangdong Provincial Laboratory of Chemistry and Fine Chemical Engineering Jieyang Center, Guangdong University of Technology, Jieyang 515200, China

**Keywords:** 3D printing, low shrinkage, hard silicone resins, UV-cured, transparent

## Abstract

Acrylated silicone elastomers for UV-curing 3D printing have gathered considerable attention in biomedical applications due to their exceptional mechanical and thermal stability. However, traditional manufacturing methods for these resins often face challenges such as stringent conditions and self-polymerization. In this study, various acrylate silicone resins (LMDT-AE) and silicone oils (PDMS-AE) were synthesized through ring-opening hydrolysis-polycondensation. The structures of LMDT-AE and PDMS-AE, with varying AE contents (molar ratio of organic groups to silicon atoms), were characterized using FTIR, ^1^H NMR, ^13^C NMR, and GPC. Additionally, their physical properties, including viscosity, density, refractive index, and transparency, were thoroughly examined. The 3D-AE silicone resin composed of LMDT-AE-2.0 and PDMS-AE-20/1, in a mass ratio of 2:1, demonstrated superior mechanical properties, thermal stability, and curing shrinkage rate compared to other formulations. This curing silicone resin is capable of producing 3D physical entities with smooth surfaces and well-defined contours. It is shown that the successful preparation of transparent and high-strength UV-cured silicone resin based on free radical polymerization can provide a potential path for high-precision biological 3D printing.

## 1. Introduction

UV-cured 3D printing technology is a rapid prototyping method that utilizes liquid photosensitive materials [1,2,3]. As a representative of photopolymerization processes, this technology relies on precisely controlled ultraviolet light to trigger crosslinking reactions in photopolymers such as acrylates or epoxy resins [4,5]. The layer-by-layer curing mechanism enables the transformation of liquid monomers into solid structures with micron-level resolution [6,7]. It offers several advantages, including low energy consumption, excellent surface quality, high precision, etc. [8,9,10]. Complex parts with intricate structures can be produced, making it challenging to manufacture traditional methods, especially for the production of complex and high-precision human organs [11,12,13,14].

Silicone resins, particularly poly(dimethylsiloxane) (PDMS)-based materials, have been widely recognized for their unique properties, including high chemical stability, thermal resistance, and excellent mechanical flexibility [15,16,17]. However, conventional silicone resins are typically cured through thermal or two-part mixing processes, which are not compatible with UV-cured 3D printing [18,19]. Recently, advancements in UV-curable silicone have opened new avenues for developing UV-cured 3D printing technology, for instance, the radical photopolymerization of acrylate-functional silicones [20,21,22], the thiol-ene photopolymerization of thiol-functional silicones [23,24,25,26], and the cationic photopolymerization of epoxy-functional silicones [27,28,29]. Therefore, acrylate-functional silicones have the advantages of a fast polymerization rate, controllable structure, low cost, simple preparation process and good oxygen resistance, which makes them a great application prospect in UV-cured 3D printing [30,31]. However, the reported acrylate-functional silicones often suffer from several drawbacks, one of the most prominent being high shrinkage during the curing process. Shrinkage can lead to significant dimensional inaccuracies in the printed objects, resulting in parts that do not meet the desired specifications [32,33]. This is particularly problematic when high-precision components are required, such as in microfluidic devices, optical lenses, and intricate molds. Additionally, the transparency of 3D-printed silicone parts is crucial for applications in optics, where clear and distortion-free materials are essential for light transmission and imaging [34]. Many existing UV-curing silicone resins either lack sufficient transparency or experience a degradation in mechanical strength after curing, further limiting their utility in these fields [26,35,36].

To address these challenges, the study of low-shrinkage transparent UV-cured 3D printing hard silicone resins has emerged as a promising research direction. With this goal in mind, this work focuses on designing and synthesizing three-dimensional reticulated crosslinked low-shrinkage acrylate silicone resins based on radical photopolymerization. A series of UV-active silicone resins, LMDT-AE, with varying acrylate double bond (AE) contents, and silicone oils, PDMS-AE, were prepared using the ring-opening hydrolysis-polycondensation method. The effects of different structures on double bond conversion rate, mechanical properties, thermal stability, and curing shrinkage rate were investigated, resulting in the development of excellent 3D printing materials capable of producing smooth-surfaced and clearly contoured 3D printed objects.

## 2. Experimental Section

### 2.1. Materials

Methacryloxy-propyl-methyl-trimethoxysilane (KH570), methacryloxy-propyl-methyl-dimethoxy-methylsilane (KH571), hexamethyldisiloxane (MM), and dimethyl cyclic siloxane (DMC) were obtained from Guangzhou Chemical Reagent Co., Ltd., Guangzhou, China. Trifluoromethanesulfonic acid (TFSA), tetramethylammonium hydroxide (TMAH), and 2,4,6-trimethylbenzoyl phosphonic acid ethyl ester (TPO-L) were sourced from Macklin Co., Ltd. (Shanghai, China). All other chemicals were purchased from commercial suppliers and used without additional purification.

### 2.2. Preparation of LMDT-AE

**Scheme 1:**
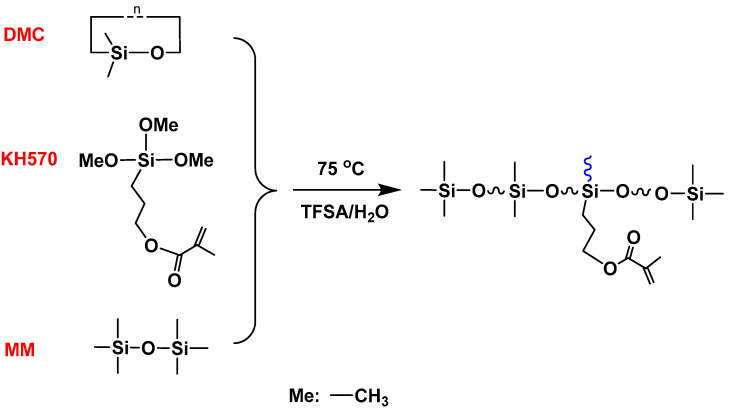
The synthesis route of LMDT-AE resins.

Specific quantities of KH570, DMC, and MM (as detailed in Table 1) were added to a four-necked flask equipped with a mechanical stirrer, condenser, thermometer, and dropping funnel. TFSA and deionized water were introduced dropwise and allowed to react at 75 °C for 3 h. After cooling the mixture to room temperature, it was washed three times with a saturated sodium carbonate-ethanol-water solution. The transparent viscous liquid resin was then obtained through vacuum distillation at 120 °C. The four LMDT-AE samples were systematically designated as LMDT-AE-2.14, LMDT-AE-2.00, LMDT-AE-1.89, and LMDT-AE-1.80 according to their respective molar ratios of organic groups to silicon atoms in the resin structure (*R* values), as quantitatively detailed in Table 1. The synthesis route is illustrated in Scheme 1.

### 2.3. Preparation of PDMS-AE

**Scheme 2:**
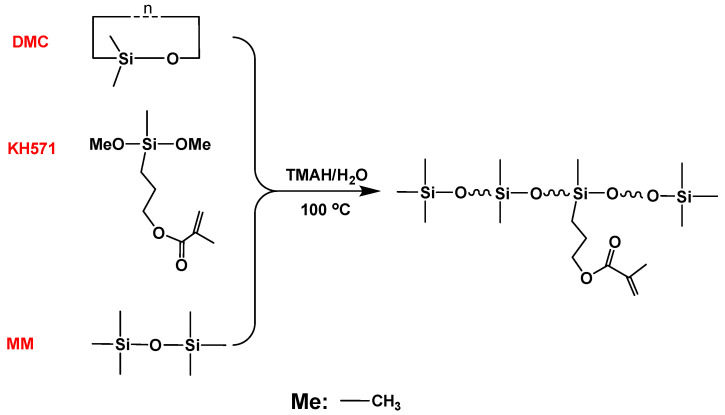
The synthesis route of PDMS-AE resins.

Similarly, various amounts of KH571, DMC, and MM (as specified in Table 2) were added to a four-necked flask with a mechanical stirrer, condenser, thermometer, and dropping funnel. TMAH and deionized water were added dropwise and reacted at 75 °C for 3 h, followed by stirring at 100 °C for 4 h until the mixture became transparent. The transparent viscous liquid resin was obtained after vacuum distillation at 160 °C. Based on the different molar ratios of DMC to KH571, as shown in Table 2, the four PDMS-AE samples were labeled as PDMS-AE-5/1, PDMS-AE-10/1, PDMS-AE-20/1, and PDMS-AE-30/1. The synthesis route is illustrated in Scheme 2.

### 2.4. Preparation of 3D Printed Silicone Resin (3D-AE)

**Scheme 3:**
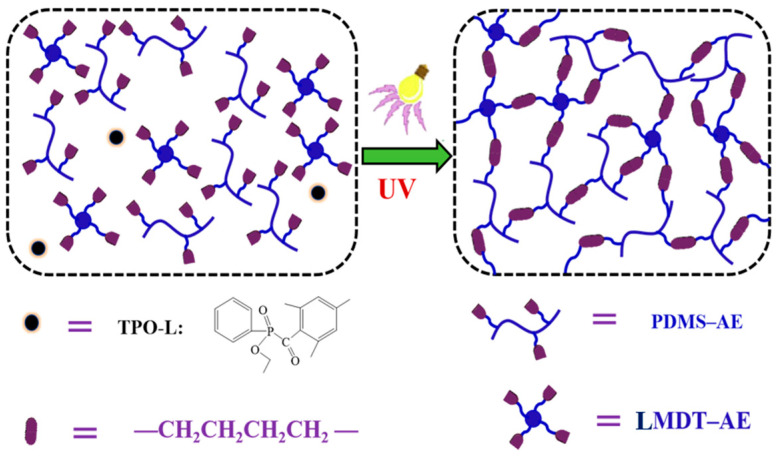
Schematic illustrations for the photo-crosslinking process of 3D-AE.

Different proportions of LMDT-AE, PDMS-AE, and TPO-L, which were selected for their UVA absorption (365~405 nm), enabling direct compatibility with UV LED curing systems, were thoroughly mixed using a vortexer in the dark (as listed in Table S1). Irgacure 1173 necessitates UVC sources or co-initiators, complicating formulations and exacerbating post-cure yellowing and therefore, TPO-L was added at 1 wt.% of the total resin mass, a concentration optimized to achieve rapid curing under 365 nm UV LED light while minimizing residual initiator and yellowing. The mixture was poured into a Teflon mold and exposed to UV radiation in a 2 kW Ultraviolet crawler UV-curing machine (UV102, Dongguan Youwei Photoelectric Co., Ltd., Dongguan, China) at 25 °C. The radiant exposure was set to 200 mJ/cm^2^, as measured by a UV energy meter (UV-int 150, UV DESIGN, Diez, Germany). The maximum intensity of 365 nm at the sample position was 70 mW/cm^2^, measured by a UV-A Radiometer (LS125, Shenzhen Linshang Technology Co., Ltd., Shenzhen, China). The 365 nm UV-curing machine was selected to align with TPO-L’s absorption profile, ensuring efficient radical generation. While 385/405 nm sources are common in commercial printers, our focus on maximizing cure efficiency for high-crosslink-density silicones necessitated this wavelength-specific optimization. The photo-crosslinking process of 3D-AE is illustrated in Scheme 3.

## 3. Results and Discussion

### 3.1. Characterization and Properties of LMDT-AE

**Figure 1:**
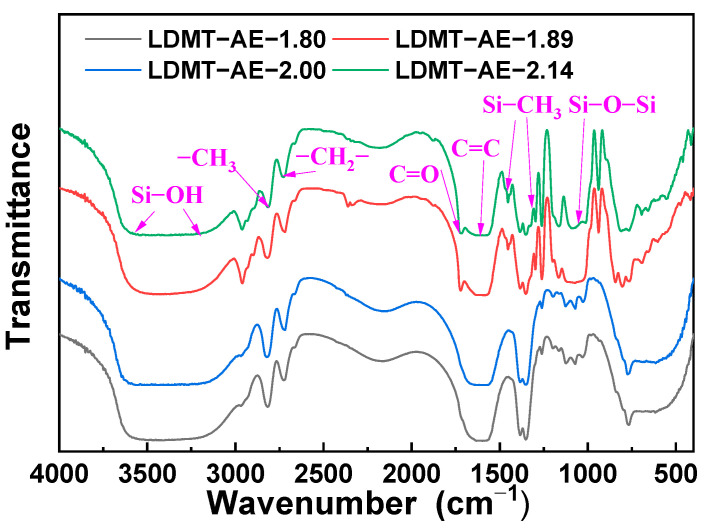
FTIR spectra of various LMDT-AE. (For clarity of presentation, the transmission spectra of LMDT-AE are shifted vertically relative to each other).

The varying content of acrylic ester C=C bonds in LMDT-AE was characterized by FITR, as shown in Figure 1. The peak around 3238–3606 cm^−1^ corresponds to the Si-OH stretching vibration absorption peak. The peak at 2839 cm^−1^ is attributed to the C-H stretching vibration absorption peak of -CH_3_. The absorption peak of C-H stretching vibration of -CH_2_- is observed at 2721 cm^−1^. The characteristic peak at 1730 cm^−1^ is attributed to ester carbonyl C=O. The C=C characteristic peak is located at 1640 cm^−1^. The continuous peaks at 1460 cm^−1^, 1265 cm^−1^ and 1184~1000 cm^−1^ are the characteristic absorption peaks of functional groups such as Si-CH_3_ and Si-O-Si. Thus, the successful synthesis of LMDT-AE resin is confirmed through FTIR characterization [37]. The spectral differences between LMDT-AE-2.14, LMDT-AE-1.89 and LMDT-AE-2.00, LMDT-AE-1.80 are attributed to variations in siloxane condensation degree and acrylate self-polymerization during synthesis.

The ^1^H NMR spectra presented in Figure S1 further confirm the structures of the four LMDT-AE samples synthesized via the ring open hydrolysis-condensation method. The ^1^H NMR data (400 MHz, CDCl_3_) are as follows: δ (ppm) = 7.26 (s, H, CDCl_3_), 6.10 and 5.52 (*dd*, 2H, =CH_2_), 4.14 (*m*, 2H, O=C-O-CH_2_), 3.84 (s, H, Si-OH), 3.57 (d, 8H, 1,4-dioxane), 1.97 (m, 3H, =C-CH_3_), 1.25 (m, 2H, -CH_2_-), 0.71 (m, 2H, SiCH_2_-), −0.02~0.25 (m, 3H, Si-CH_3_). The distinct methacryloyloxypropyl and Si-CH_3_ proton peaks observed in the spectra indicate the successful synthesis of the LMDT-AE resin. Additionally, the ^13^C NMR spectrum reveals a shift in the Si-CH_3_ peaks to 0.80 and 2.20 ppm after ring-opening hydrolysis-condensation in DMSO. The absence of Si-OMe (-OMe denotes a methoxy group) signals in the reactants, as shown in Figure S2, confirms that Si-OMe is completely hydrolyzed and condensed into the LMDT resin structure.

Additionally, the content of acrylic ester double bond (AE) in the four LMDT-AE resins was calculated, as detailed in Table S2. The measured AE content of these resins closely aligns with the theoretical values, with only minor discrepancies. The errors of the four LMDT-AE resins are 2.42%, 2.67%, 2.92% and 2.95%, respectively. The error tends to increase as the molar ratio of organic groups to a silicon atom (*R* value) decreases. This can be attributed to the increasing viscosity of LMDT-AE. Higher viscosity, associated with higher molecular weight, can lead to a non-uniform molecular weight distribution, thereby increasing the errors.

**Figure 2:**
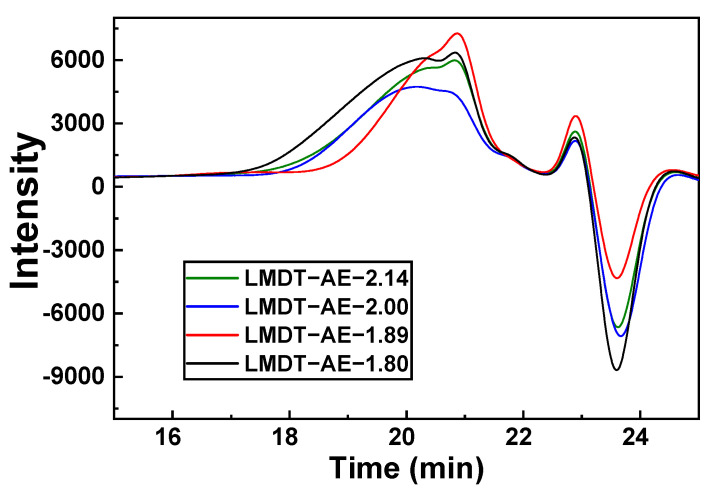
GPC curve of various LMDT-AE.

The weight average molecular weight (Mw), number average molecular weight (Mn) and dispersibility index (PDI) of LMDT-AE were determined by gel permeation chromatography (GPC). As shown in Table S3, both Mw and Mn exhibit a trend of initially decreasing and then increasing as the *R* value decreases, which correlates with the elution time depicted in Figure 2. The PDI shows a trend of first increasing and then decreasing, which is influenced by the reaction conditions and the ratio of raw materials used. Notably, LMDT-AE-2.14 has a higher molecular weight and a narrower PDI value, indicating a more uniform molecular weight distribution. This suggests that the unsaturated double bonds in the methacryloyloxypropyl group remain stable during the preparation process.

The physical properties of LMDT-AE are crucial for the performance of the subsequent 3D printed silicone resin composite, 3D-AE. These properties were evaluated as shown in Table S4 and Figure S3. All prepared LMDT-AE samples are colorless and transparent viscous substances. Samples with varying *R* values are low viscosity, high transparency viscous liquids exhibiting distinct viscosity gradients. As the *R* value decreases, a wave-like trend is observed in their physical properties, such as viscosity, density, refractive index and transmittance at 450 nm. This can be attributed to the reaction conditions and the ratio of raw materials used. Among these, LMDT-AE-2.14 demonstrates superior physical properties owing to its high molecular weight and excellent uniformity in molecular weight distribution.

**Figure 3:**
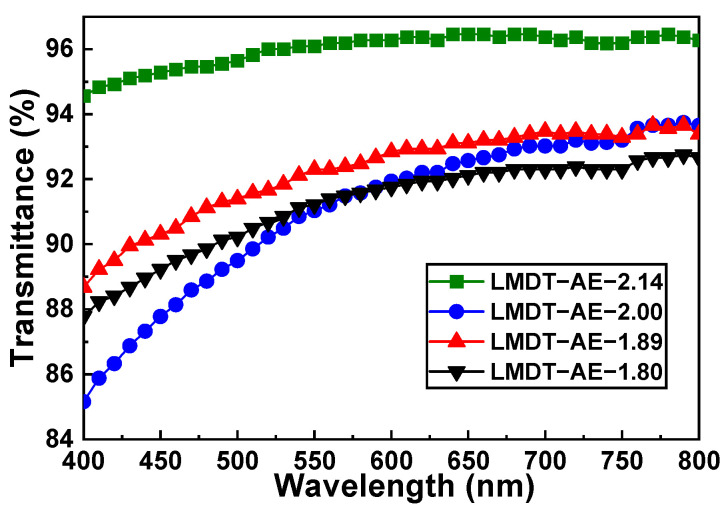
Light transmittance of different LMDT-AE. (Sample thickness is 10.0 mm).

The visible light transmittance of the four different LMDT-AE structures is presented in Figure 3. The transmittance of LMDT-AE is closely related to its viscosity and transparency. As the *R* value decreases, the visible light transmittance of LMDT-AE at 400–800 nm shows a slight downward trend. This could be ascribed to the increased difficulty of homopolymerization with higher molecular weight, which results in an increased PDI value, thereby reducing the visible light transmittance.

### 3.2. Characterization and Properties of PDMS-AE

**Figure 4:**
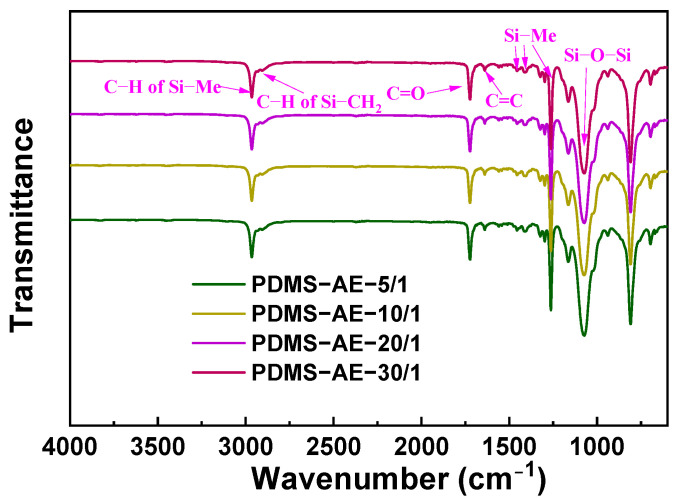
FTIR spectrum of PDMS-AE. (For clarity of presentation, the transmission spectra of PDMS-AE are shifted vertically relative to each other).

From the FTIR spectrum of PDMS-AE (Figure 4), the C-H stretching vibration absorption peaks for the methyl (-CH_3_) and methylene (-CH_2_-) groups in Si-Me and Si-CH_2_CH_2_CH_2_- respectively are observed at 2963 cm^−1^ and 2895 cm^−1^, respectively. The characteristic peak for the ester carbonyl C=O bond in the acrylic ester groups is located at 1725 cm^−1^. The absorption peak for the unsaturated bond C=C in the acrylic ester group is located at 1643 cm^−1^. The characteristic peaks for Si-Me are found near 1450 cm^−1^, 1415 cm^−1^ and 1265 cm^−1^. The continuous peaks in the range of 950~1210 cm^−1^ are indicative of the linear Si-O-Si structure [37]. The presence of distinct characteristic absorption peaks for Si-O-Si, Si-Me, Si-CH_2_-, C=O and acrylate C=C confirms their successful incorporation. Additionally, the absence of any broad or flat peaks above 3400 cm^−1^ indicates that ring-opening hydrolysis-copolymerization is complete and that the Si-OH bonds have fully condensed. This confirms the successful synthesis of PDMS-AE. Unlike acid-catalyzed processes that induce self-polymerization of unsaturated bonds in acrylate groups, TMAH-catalyzed ring-opening copolymerization minimizes unsaturated bond addition. Consequently, all four PDMS-AE variants exhibit stable characteristic absorption peaks of unsaturated bonds at approximately 1643 cm^−1^. The spectral similarity across samples reflects the homogeneity of PDMS-AE synthesized via TMAH-catalyzed ring-opening copolymerization compared with acid-catalyzed hydrolysis.

The structure of PDMS-AE was further confirmed through ^1^H NMR spectra (Figure S4). The ^1^H NMR data (400 MHz, CDCl_3_) are as follows: δ (ppm) = 6.10 and 5.81 (**d**, H, =CH_2_), 4.09 (m, 2H, O=C-O-CH_2_), 3.68 (d, 8H, dioxane), 1.99 (m, 3H, =C-CH_3_), 1.66 (m, 2H, -CH_2_-), 0.52 (m, 2H, SiCH_2_-), −0.15~0.19 (m, 3H, Si-CH_3_). The presence of significant methyl methacrylate groups and hydrogen proton peak of Si-CH_3_ further confirm the successful preparation of PDMS-AE.

Additionally, the content of acrylic ester double bonds (AE) in the four PDMS-AE resins was calculated, as detailed in Table S5. The measured AE content of the four PDMS-AE resins is close to the theoretical values, with only minor discrepancies. The errors for the four PDMS-AE resins are 4.78%, 4.44%, 4.00%, and 3.61%, respectively. The error decreases as the molar ratio of dimethyl chain links (D chain links) to methyl methacrylate propyl (AE chain links) increases. This is related to the viscosity of PDMS-AE and the content of AE chain links. A higher AE chain link content leads to a more significant influence of preparation process factors, resulting in an increase in AE content error.

**Figure 5:**
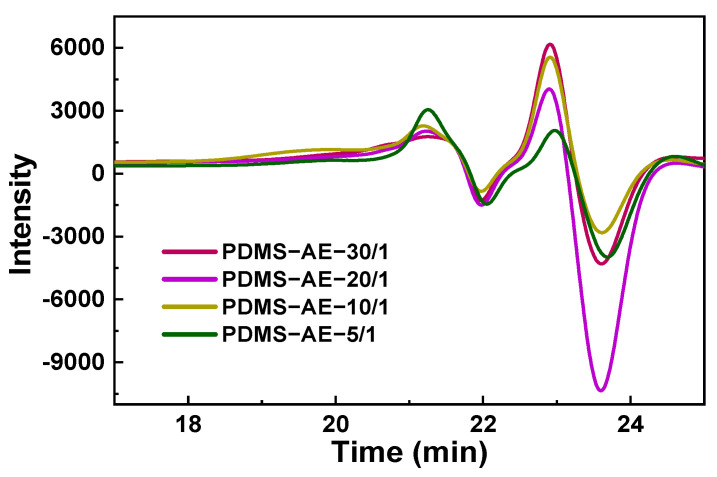
GPC curve of various PDMS-AE.

Table S6 summarizes the molecular weights and PDI values of different PDMS-AE samples. As the molar ratio of D chain links to AE chain links increases, both the weight average molecular weight (*M*w) and number average molecular weight (*M*n) show an increasing trend, which corresponds to the elution time depicted in Figure 5. The molecular weight distribution of PDMS-AE is uniform, with a low PDI. The unsaturated double bond in the methacryloxypropyl group remains stable during the preparation process, significantly reducing the likelihood of gel formation due to detachment or self-polymerization. This finding further substantiates that the high degree of similarity in PDMS-AE’s infrared spectra is attributable to the stable presence of unsaturated bonds along the polysiloxane chains.

**Figure 6:**
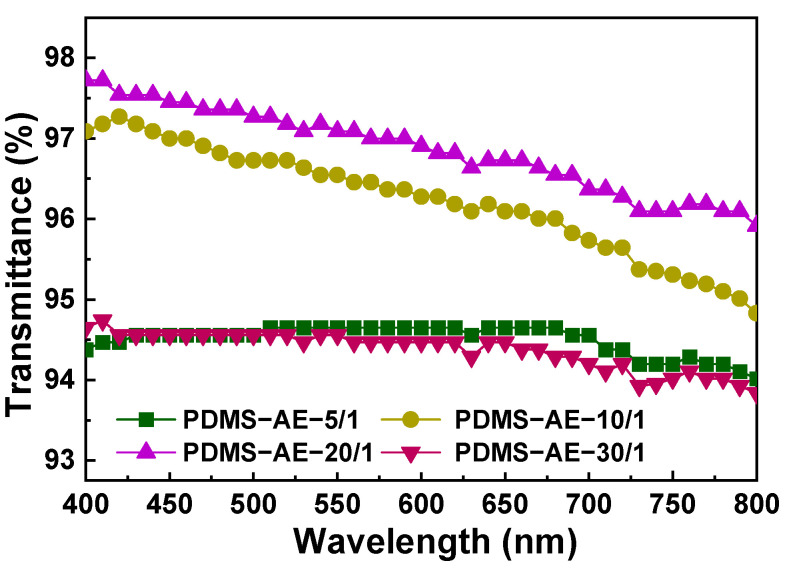
Light transmittance of different PDMS-AE. (Sample thickness is 10.0 mm).

The physical properties of the four different PDMS-AE samples were also evaluated (Table S7 and Figure S5). All prepared PDMS-AE samples are colorless and transparent viscous liquids. As the molar ratio of D chain links to AE chain links increases, the viscosity tends to rise while the density and refractive index decrease. It was observed that when the molar ratio of D chain links to AE chain links exceeds 20/1, the transmittance at 450 nm begins to decrease. This is attributed to the ratio of raw materials, as well as the steric hindrance effect and thermal stability of the methacryloyloxypropyl group. Consequently, PDMS-AE-20/1 can be selected as the optimal photoactive silicone oil for subsequent formulations, which can be used to prepare a 3D-printed silicone resin composite, 3D-AE, with enhanced performance. The visible light transmittance of the four different PDMS-AE structures is presented in Figure 6. Among them, PDMS-AE-20/1 exhibits the highest light transmittance, which can be attributed to its balanced viscosity and uniform molecular weight distribution.

### 3.3. UV-Curing Kinetics of 3D-AE Elastomers

**Figure 7:**
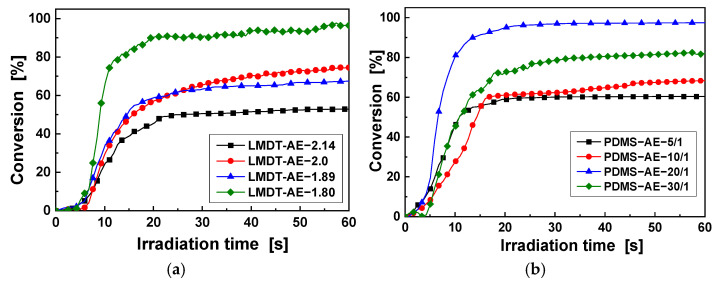
Conversion curves of double bonds of LMDT-AE (**a**) and PDMS-AE (**b**).

The photopolymerization conversion of acrylic ester double bonds was measured using real-time infrared spectra on a Nicolet iS50 spectrometer equipped with a UV radiation light source. The conversion rates of LMDT-AE and PDMS-AE were calculated according to Equation (S2). As shown in Figure 7a, LMDT-AE-1.80 demonstrated the highest double bond conversion rate among the four different LMDT-AE structures, reaching 93.3% within 25 s. This can be attributed to its high AE content. Under UV radiation, a dense curing film forms rapidly on the surface, which prevents oxygen inhibition of polymerization and enhances the double bond conversion rate.

For PDMS-AE, each of the four different structures achieved their respective highest double bond conversion within 15 s, as shown in Figure 7b. PDMS-AE-20/1 exhibited the highest double bond conversion rate among the four structures, reaching 98.8%. This can be attributed to its uniform molecular weight distribution. The surface crosslinking network effectively prevents oxygen inhibition of polymerization without hindering the deep penetration of UV light.

Three-dimensional-printed silicone resins (3D-AE) were prepared using varying proportions of LMDT-AE and PDMS-AE in the presence of a photo-initiator. Figure S6 displayed the double bond conversion rate curves for 3D-AE when the mass ratio of LMDT-AE to PDMS-AE was 2:1. Regardless of the AE content in LMDT when LMDT-AE was combined with PDMS-AE-20/1 (with a molar ratio of D chain links to AE chain links of 20:1), the resulting 3D-AE achieved a higher double bond conversion rate than other combinations (Figure S6). This can be attributed to the uniform structure and molecular weight distribution of PDMS-AE-20/1. Among the different formulations, LMDT-AE-2.0 and PDMS-AE-20/1 (with a mass ratio of 2:1) exhibited the best conversion rate under the same conditions, reaching 95.6%. This is likely due to the crosslinking density of the system and the uniformity of surface crosslinking.

**Figure 8:**
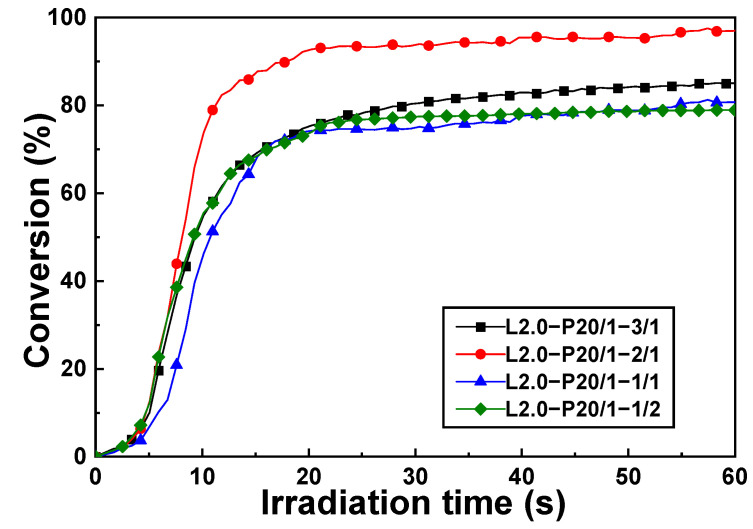
The double bond conversion curves of 3D-AE were prepared from different mass ratios of LMDT-AE and PDMS-AE.

Furthermore, the effect of the mass ratio of LMDT-AE to PDMS-AE on the conversion of acrylic ester double bonds was explored, as shown in Figure 8. When the mass ratio of LMDT-AE to PDMS-AE was 3:1, the conversion rate was lower than that of the 2:1 ratio. The higher content of LMDT-AE initially led to a high surface crosslinking density, which reduced the deep penetration of UV light, although it also decreased the oxygen inhibition effect. Conversely, when the mass ratio of LMDT-AE to PDMS-AE was less than 2:1, the low crosslinking density resulted in a significant oxygen inhibition effect, which adversely affected the UV photopolymerization process.

### 3.4. Mechanical Properties of 3D-AE Elastomers

The stress-strain properties of 3D-AE prepared with different PDMS-AE formulations were investigated, as shown in Figure S7, with a mass ratio of LMDT-AE to PDMS-AE of 2:1. The tensile strength initially increased and then decreased with the increasing molar ratio of D chain links to AE chain links in PDMS-AE, while the elongation at break consistently increased. Notably, the tensile strength of 3D-AE prepared using PDMS-AE-20/1 was higher than that of other PDMS-AE formulations with varying molar ratios of D chain links to AE chain links. This suggests that tensile strength is closely related to the UV photopolymerization behavior, PDMS-AE structure, and crosslinking degree.

The tensile strengths of 3D-AE prepared by mixing LMDT-AE-2.14, LMDT-AE-2.0, LMDT-AE-1.89, and LMDT-AE-1.80 with PDMS-AE-20/1 were 3.18 MPa, 3.85 MPa, 2.88 MPa, and 1.88 MPa, respectively. The corresponding elongations at break were 3.54%, 3.78%, 3.20%, and 3.61%. Among these formulations, the combination of LMDT-AE-2.0 and PDMS-AE-20/1 (with a mass ratio of 2:1) exhibited the best tensile strength and elongation at break.

**Figure 9:**
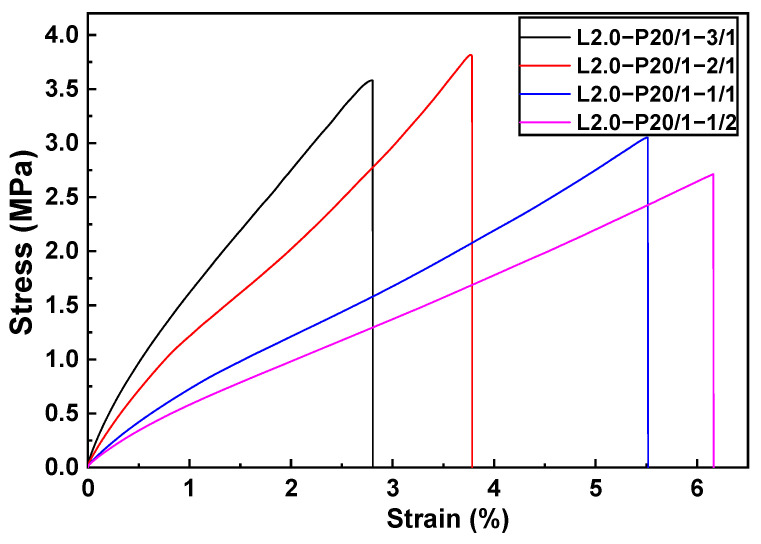
The stress-strain curve of 3D-AE was prepared from different mass ratios of LMDT-AE and PDMS-AE.

Furthermore, the impact of the mass ratio of LMDT-AE to PDMS-AE on the tensile strength and elongation at break was examined, as illustrated in Figure 9. When the mass ratio of LMDT-AE to PDMS-AE was 3:1, both the tensile strength and elongation at the break of the 3D-AE were lower compared to a ratio of 2:1. Thus, appropriately reducing the content of LMDT-AE can enhance the mechanical properties of 3D-AE. Conversely, when the mass ratio of LMDT-AE to PDMS-AE was less than 2:1, the lower crosslinking density led to a significant oxygen inhibition effect, which adversely affected the UV photopolymerization process. The reduced content of the reinforcing resin resulted in a lower crosslinking density, thereby diminishing its mechanical strength. Optimal mechanical properties of 3D-AE were achieved at a mass ratio of LMDT-AE to PDMS-AE of 2:1, with a tensile strength of 3.85 MPa and an elongation at a break of 3.78%.

### 3.5. Thermal Properties of 3D-AE Elastomers

The thermal stability of 3D-AE is closely related to the crosslinking density and the temperature resistance of the structures of PDMS-AE and LMDT-AE. Thermogravimetric analysis (TGA) was employed to assess the thermal stability and decomposition process of 3D-AE. The decomposition temperature (T5%), defined as the temperature at which a 5 wt% weight loss occurs, was used to characterize the strength of its heat stability. The stress-strain properties of 3D-AE prepared with different PDMS-AE formulations were explored as shown in Figure S8, with a mass ratio of LMDT-AE to PDMS-AE of 2:1. The T_5%_, T_50%_, Tmax, and residual rate of 3D-AE prepared using PDMS-AE-20/1 are relatively high, which can be attributed to the structure, molecular weight distribution, and crosslinking degree of PDMS-AE system (Figure S8 and Table S8). Compared to other formulations, LMDT-AE-2.0 and PDMS-AE-20/1 (with a mass ratio of 2:1) exhibited the best thermal stability. This is related to the structure and crosslinking density of the LMDT-AE-2.0 and PDMS-AE-20/1 formulation.

**Figure 10:**
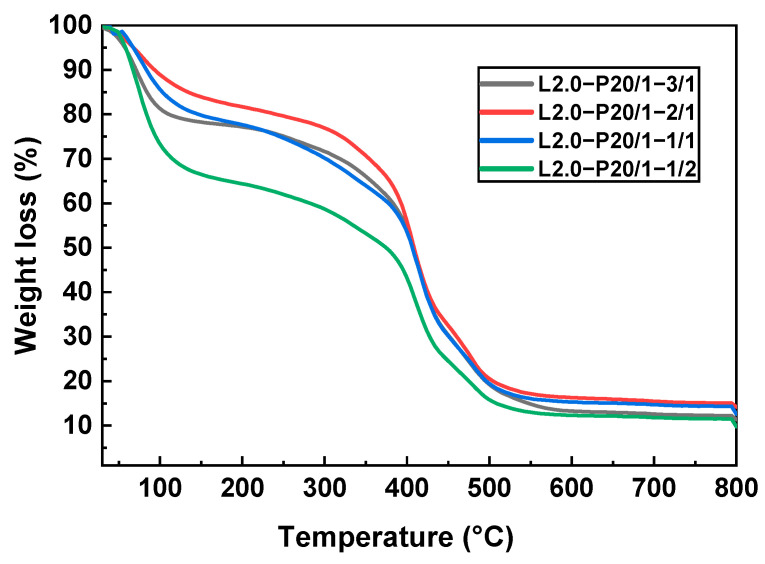
TGA curves of 3D-AE were prepared from different mass ratios of LMDT-AE and PDMS-AE.

Furthermore, the effect of the mass ratio of LMDT-AE to PDMS-AE on thermal stability was investigated, as shown in Figure 10. When the mass ratio of LMDT-AE to PDMS-AE was 3:1, the thermal stability of the 3D-AE was inferior to that of the 2:1 ratio. This is because the thermal stability of 3D-AE is closely tied to the crosslinking density. Appropriately reducing the mass ratio of LMDT-AE can help balance the deep penetration of UV light and the surface oxygen-blocking effect. When the mass ratio of LMDT-AE to PDMS-AE was less than 2:1, the low crosslinking density reduced their thermal stability.

Subsequently, the DSC curves of 3D-AE prepared by different PDMS-AE formulations were obtained, as shown in Figure S8, with a mass ratio of LMDT-AE to PDMS-AE of 2:1. The glass transition temperature (Tg) of 3D-AE prepared using PDMS-AE-20/1 was higher than that of other PDMS-AE formulations with varying molar ratios of D chain links to AE chain links. Compared to other formulations, LMDT-AE-2.0 and PDMS-AE-20/1 (with a mass ratio of 2:1) exhibited the highest Tg. This can be attributed to the structure and crosslinking density of LMDT-AE-2.0 and PDMS-AE-20/1 formulation.

**Figure 11:**
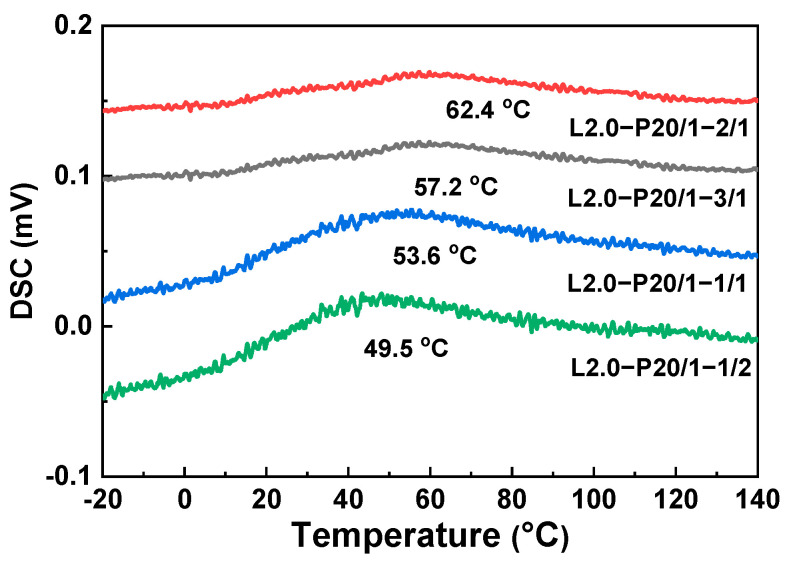
DSC curves of 3D-AE were prepared from different mass ratios of LMDT-AE and PDMS-AE (The experimental uncertainty is ±0.1 °C).

Additionally, the effect of the mass ratio of LMDT-AE to PDMS-AE on Tg was explored, as shown in Figure 11. As the mass ratio of LMDT-AE to PDMS-AE decreases, Tg initially increases and then decreases, which aligns with the trends observed in double bond conversion rate, tensile strength, and thermal stability. The highest Tg of 3D-AE was achieved when the mass ratio of LMDT-AE to PDMS-AE was 2:1, with a Tg of 62.4 °C.

### 3.6. Curing Shrinkage Properties of 3D-AE Elastomer

The curing shrinkage rate is a critical factor for 3D printing materials, as it can significantly impact the accuracy and performance of 3D printed objects. Figure S10 shows the curing shrinkage rate of 3D-AE prepared with a mass ratio of LMDT-AE to PDMS-AE of 2:1. With the structure of LMDT-AE resin remaining constant, the curing shrinkage tends to decrease as the molar ratio of D chain to AE chain increases. Moreover, when the molar ratio of D chain to AE chain segments exceeds 20/1, the rate of shrinkage reduction begins to slow down. This is related to the AE content in PDMS-AE. Lower AE content means fewer reactive sites per unit volume of monomer, resulting in a lower curing shrinkage rate.

Additionally, it has been observed that when the structure of PDMS-AE remains unchanged, the shrinkage rate tends to increase as the *R* value in LMDT-AE increases. This is because a decrease in the *R* value leads to a higher content of AE in LMDT, thereby reducing the curing shrinkage rate. Consequently, using LMDT-AE-2.0 and PDMS-AE-20/1 in the preparation of 3D-AE results in a relatively low curing shrinkage rate.

**Figure 12:**
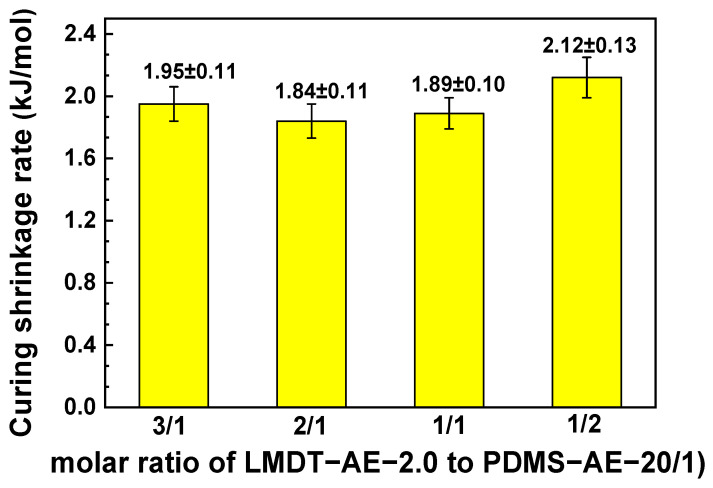
Curing shrinkage rates of 3D-AE prepared with different mass ratios for LMDT-AE and PDMS-AE.

Figure 12 illustrates the curing shrinkage curves of 3D-AE prepared with different mass ratios of LMDT-AE to PDMS-AE. As seen in Figure 12, the curing shrinkage rate initially decreases and then increases as the mass ratio of LMDT-AE to PDMS-AE decreases. This is because LMDT-AE has a microspherical structure, and reducing its amount effectively lowers the AE content in the monomer volume, thereby reducing the curing shrinkage rate. When the mass ratio of LMDT-AE to PDMS-AE is reduced below 2:1, the hardness of the cured 3D-AE decreases, and its flexibility increases. The AE photopolymerization can drive the movement of PDMS molecular chains, causing the curing system’s shrinkage rate to rise again. Therefore, when the mass ratio of LMDT-AE-2.0 to PDMS-AE-20/1 is 2:1, the comprehensive performance of 3D-AE is optimal, with a lower curing shrinkage rate of 1.84 ± 0.10%. This shrinkage rate is below 2%, meeting the requirements for shrinkage in 3D printing.

### 3.7. Photo-Patterning and 3D Printing

**Figure 13:**
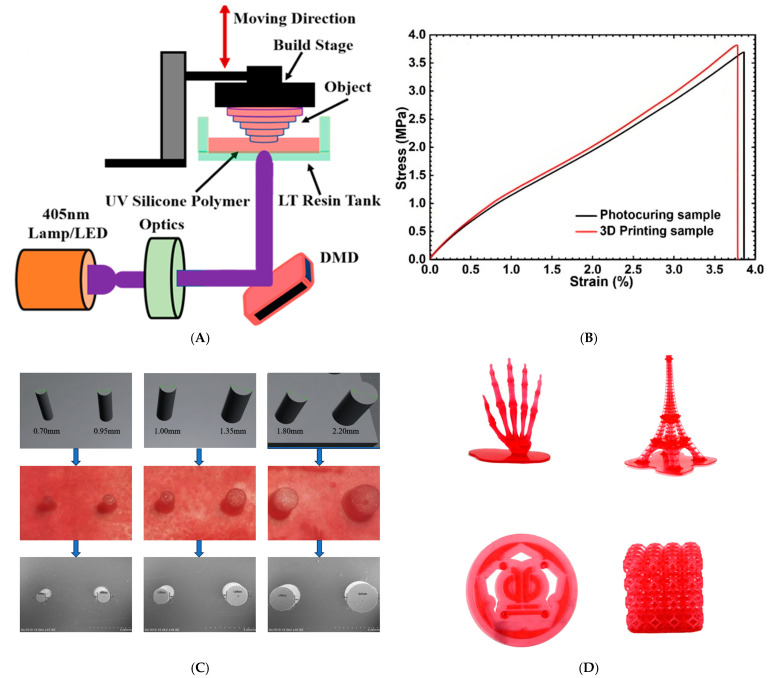
The cured samples and effects of L2.0-P20/1-2/1 formula. (**A**) Schematic principle of LCD-based 3D printing; (**B**) Stress-strain curves of 3D printing and traditional direct UV cured dumbbell shaped splines; (**C**) Accuracy test pattern of 3D-printing; (**D**) Various printing entities printed by adding red color paste to the L2.0-P20/1-2/1 formula (“bones of hand”, “Eiffel Tower”, “emblem of the Guangdong University of Technology”, “hollow cage-shaped cubes”).

The patterns were prepared using a layered stacking method on a LCD desktop 3D printer (HALOT-ONE Pro, Shenzhen Creality 3D Technology Co., Ltd., Shenzhen, China). The printing parameters were set as follows: a print thickness of 0.05 mm per layer, an exposure time of 30 s, and an initial five layers on the bottom with an exposure time of 30 s and a delayed exposure of 15 s. The 3D-AE silicone elastomer, with a ratio of LMDT-AE-2.0 to PDMS-AE-20/1 of 2:1 and containing 0.1 wt% red pigment (Orasol Red 355, BASF SE, Ludwigshafen, Germany) for clearer pattern visibility, was irradiated at 30 mW/cm^2^ for 30 s per layer. The cured samples were then rinsed with ethanol to remove any uncured components (Figure 13A).

As shown in Figure 13B, the tensile strength and elongation at the break of the 3D-printed dumbbell-shaped specimens are essentially consistent with those obtained through traditional bulk polymerization. This indicates that objects formed via the layer-by-layer stacking mechanism of 3D printing possess similar properties to those formed by bulk polymerization, demonstrating strong molecular-level bonding between the printed layers.

Pigments were added to the resin formulation to enable in situ coloration during printing, avoiding post-curing surface treatments that compromise transparency. This approach ensures uniform color distribution, which is critical for optical applications such as lenses or light-guiding components. Figure 13D illustrates that the addition of red organic pigment to the L2.0-P20/1-2/1 formulation does not affect its UV curing rate, allowing for the successful printing of 3D entities with smooth surfaces and clear contours, such as the emblem of Guangdong University of Technology, the Eiffel Tower, a hollow cage-shaped cube, and a hand bone. The inclusion of pigments demonstrates the resin’s adaptability to commercial workflows, with no adverse effects on curing kinetics or tensile strength, as shown in Figure 13B.

To test the accuracy of 3D printing, a cylindrical precision 3D model was specifically designed, as shown in Figure 13C. Small cylinders with various diameters: 0.70 mm, 0.95 mm, 1.00 mm, 1.35 mm, 1.80 mm, and 2.20 mm, were included in the design. After characterization using SEM (Scanning Electron Microscopy), the diameters of the small cylinders in the 3D-printed objects matched the designed diameters with minimal error (Table S9). This indicates that 3D-AE silicone elastomer can be used to print physical objects with high accuracy, offering potential applications in biological 3D printing.

## 4. Conclusions

Transparent acrylate silicone resins (LMDT-AE) and silicone oils (PDMS-AE) with varying structures were synthesized using the open-loop hydrolysis-condensation method. The structures of LMDT-AE and PDMS-AE, with different AE contents, were characterized using FTIR, ^1^H NMR, ^13^C NMR, and GPC. Additionally, the viscosity, density, refractive index, and transmittance of these materials were tested. The double bond conversion rates of LMDT-AE and PDMS-AE, along with their combinations in various mass ratios, were also explored. The optimized 3D-AE silicone resin, with a mass ratio of LMDT-AE-2.0 to PDMS-AE-20/1 of 2:1, demonstrated superior mechanical properties, thermal stability and curing shrinkage rate. It exhibited a tensile strength of 3.85 MPa, elongation at break of 3.78%, curing shrinkage of 1.84%, T_5%_ of 78.3 °C, T_50%_ of 401.5 °C, and a glass transition temperature (Tg) of 62.4 °C. This silicone resin is capable of producing 3D physical entities with smooth surfaces and clear contours, offering a simple and effective approach for high-accuracy biological 3D printing.

## Figures and Tables

**Table 1:** Raw material and dosage for synthesizing LMDT-AE.

Name	KH570	DMC	MM	TFSA ^1^	H_2_O ^2^	*R*
LMDT‒AE-2.14	24.84 g (0.10 mol)	29.60 g (0.40 mol)	16.2 g (0.10 mol)	0.35 g	4.32 g	2.14
LMDT‒AE-2.00	49.68 g (0.20 mol)	29.60 g (0.40 mol)	16.2 g (0.10 mol)	0.48 g	8.64 g	2.00
LMDT‒AE-1.89	74.52 g (0.30 mol	29.60 g (0.40 mol)	16.2 g (0.10 mol)	0.60 g	12.96 g	1.89
LMDT‒AE-1.80	99.36 g (0.40 mol)	29.60 g (0.40 mol)	16.2 g (0.10 mol)	0.73 g	17.28 g	1.80

^1^ Amount of TFSA: 0.50% × m_(KH570+DMC+MM)_; ^2^ Amount of H_2_O: 18 × 0.8 × 3n_KH570_.

**Table 2:** Raw material and dosage for synthesizing LMDT-AE.

Name	KH571	DMC	MM	TMAH ^1^	H_2_O ^2^
PDMS-AE-5/1	83.64 g (0.36 mol)	133.49 g (1.8 mol)	0.81 g (0.005 mol)	0.44 g	15.55 g
PDMS-AE-10/1	41.82 g (0.18 mol)	133.49 g (1.8 mol)	0.81 g (0.005 mol)	0.35 g	6.48 g
PDMS-AE-20/1	20.91 g (0.09 mol)	133.49 g (1.8 mol)	0.81 g (0.005 mol)	0.31 g	3.24 g
PDMS-AE-30/1	13.94 g (0.06 mol)	133.49 g (1.8 mol)	0.81 g (0.005 mol)	0.30 g	2.60 g

^1^ Amount of TMAH: 0.20% × m_(KH571+DMC+MM)_; ^2^ Amount of H_2_O: 18 × 0.8 × 2n_KH571._

## Data Availability

The original contributions presented in the study are included in the article; further inquiries can be directed to the corresponding authors.

## Supplementary Materials

The following supporting information can be downloaded at: https://www.mdpi.com/article/10.3390/polym17081123/s1, Figure S1: ^1^H NMR spectrum of various LMDT-AE; Figure S2: ^13^C NMR spectrum of LMDT-AE before and after reaction; Figure S3: Appearance of various LMDT-AE; Figure S4:^1^H NMR spectrum of PDMS-AE; Figure S5:Appearance of various PDMS-AE.; Figure S6: Double bond conversion curve of 3D-AE was prepared from different PDMS-AE at the mass ratio of LMDT-AE to PDMS-AE was 2/1; Figure S7: The stress-strain curve of 3D-AE was prepared from different PDMS-AE when the mass ratio of LMDT-AE to PDMS-AE was 2/1; Figure S8: The TGA curves of 3D-AE were prepared from different PDMS-AE when the mass ratio of LMDT-AE to PDMS-AE was 2/1; Figure S9: When the mass ratio of LMDT-AE to PDMS-AE was 2/1, the DSC curve of 3D-AE was prepared from different PDMS-AE; Figure S10: When the mass ratio of LMDT-AE to PDMS-AE was 2/1, curing shrinkage rate of 3D-AE was prepared from different PDMS-AE; Table S1: Formulas for 3D-printing silicone resin composition 3D-AE; Table S2: AE contents of LMDT-AE; Table S3: GPC data of LMDT-AE; Table S4: Physical properties of LMDT-AE; Table S5: AE contents of PDMS-AE; Table S6: GPC data of PDMS-AE; Table S7. Physical properties of PDMS-AE; Table S8: TGA date of various 3D-AE; Table S9: Error between the design value of cylinder diameter and the actual measurement value.
