# Supplementary material for: Low Shrinkage Transparent UV-Cured 3D Printing Hard Silicone Resins
Source: Polymers (Basel). 2025 Apr 21;17(8):1123. doi: 10.3390/polym17081123 (PMC12030683; doi:10.3390/polym17081123)
Supplement: Supplementary file 1 [file polymers-17-01123-s001.zip › polymers-3543957-supplementary.pdf]

# Low shrinkage transparent UV−cured 3D printing silicone resins

Haibo Wu[1,3], Qili Shen[1,4], Zhu Liu[2,*], Xiantai Zhou[3,*], Yanxiong Fang[1,3,*], Hongping Xiang[1], Xiangxuan Liu[1]

[1] School of Chemical Engineering and Light Industry, Guangdong University of Technology, Guangzhou 510006, P. R. China; wu.haibo08@163.com (H.W.); qlshen1216@163.com (Q.S.); xianghongping@gdut.edu.cn (H.X.); p−xxliu@gdut.edu.cn (X.L.)

[2] School of Dayawan Chemical and New Materials, School of Chemistry and Materials Engineering, Huizhou University, Huizhou, Guangdong, 516007, P. R. China

[3] School of Chemical Engineering and Technology, Sun Yat−sen University, Zhuhai 519082, P.R. China

[4] Guangdong Provincial Laboratory of Chemistry and Fine Chemical Engineering Jieyang Center, Guangdong University of Technology, Jieyang, 515200, P. R. China

*Correspondence: lz0927@hzu.edu.cn (Z.L.); zhouxtai@mail.sysu.edu.cn (X.Z.); fangyx@gdut.edu.cn (Y.F.)

## 1. Characterization

Fourier transform infrared spectroscopy (FT−IR) was recorded on a Nicolet iS50 spectrometer (Thermo Scientific) by a disc of KBr with a resolution of 4 $cm^{-1}$ over 32 scans from 4000 to 600 $cm^{-1}$. Elemental analysis (EA) was performed on Vario EL cube (Elementar company, Germany). Nuclear magnetic resonance ($^1H$ NMR) was implemented on a Bruker AVANCE III 400 MHz with 1,4−dioxane as an internal standard, $CDCl_3$ was used as the solvent. $^{13}C$ NMR was implemented on a Bruker AVANCE III 600 MHz with $CDCl_3$ as the solvent.

$^1H$ NMR internal standard method was used to test the content of unsaturated double bonds (AE%) in acrylic ester groups in PDMS−AE and LMDT−AE according to Eq. S1.

$$AE\ \% = \frac{\frac{A}{3}\times w_i}{\frac{B}{8}\times w_s\times 88} \times 100\% \qquad \text{(Eq. S1)}$$

where AE% is the content of unsaturated double bonds in 100 g PDMS−AE and LMDT−AE (mol/100 g). $w_s$ is the mass of PDMS−AE and LMDT−AE (g); $w_i$ is the mass of the internal standard 1,4−dioxane (g); the relative molecular weight of 1,4−dioxane is 88 g/mol, 8 is the number of hydrogen protons in 1,4−dioxane, 3 is the number of hydrogen protons in the unsaturated double bond; A is the peak area of hydrogen proton absorption in unsaturated double bonds, B is the peak area of hydrogen proton absorption in 1,4−dioxane.

The photopolymerization conversion and rate were determined by real−time infrared spectra (RT–IR) on Nicolet iS50 spectrometer equipped with a UV radiation light source (MUA−165, Mejiro Genossen, Japan). Using KBr tablets as the carrier, the test range is 700–4000 $cm^{-1}$. The test was carried out under 395 nm with an irradiation intensity of 60 $mW/cm^2$.

The Si−Me characteristic absorption peak at 1265 $cm^{-1}$ is used as the internal standard, and the characteristic absorption peak near 1640 $cm^{-1}$ is monitored. According to Eq. S2, the conversion rate of acrylic ester double bonds can be calculated and monitored in real time.

$$C_{AE} = \frac{A_0 - A_t}{A_0} \times 100\% \qquad \text{(Eq. S2)}$$

where, $C_{AE}$ was the conversion rate of acrylic ester double bonds (%); $A_0$ and $A_t$ were the normalized peak area of acrylic ester double bonds before and after irradiation time

*t*, respectively.

The molecular weight and polydispersity were tested in Shimadzu RID−20A gel permeation chromatograph (GPC). The mobile phase was tetrahydrofuran, and the flow rate was 1.0 mL/min. The transmittance was analyzed by UV−visible spectrophotometer (UV2450, Shimadzu, Japan) ranging from 400 to 800 nm.

The viscosity measurement was conducted using an NDJ−8S digital viscometer (Shanghai Hengping Instrument Co., Ltd., China) in compliance with national standard GB/T 10247−2008.

The liquid density was measured using a Shimadzu AUY 120 densitometer (Shimadzu Corporation, Japan) in compliance with ISO 3521 international standard. The refractive index was determined with a Shanghai Optical Instrument Factory 2WAJ Abbe refractometer following GB/T 6448−2008 national standard.

Stress−strain tensile test was carried out on a SANS CMT 6000 Universal Tester with a crosshead speed rate of 50 mm/min at 25 °C according to ASTM D412. The curing shrinkage rate test was carried out on a Fully automatic curing shrinkage tester (MAY−S3233, China) according to KSM ISO 3521−2010 (2020) standard. Thermogravimetric analysis (TGA) was performed on a TG209F1 (Netzsch, Germany) from 30 to 800 °C by 10 °C/min under $N_2$ flow of 20 mL/min. The glass transition temperature (Tg) of the sample was measured using a differential scanning calorimetry analyzer (DSC214, Netzsch, Germany) from −30 to 200 °C by 5 °C/min under $N_2$ flow of 50 mL/min.

## 2. Results and discussion

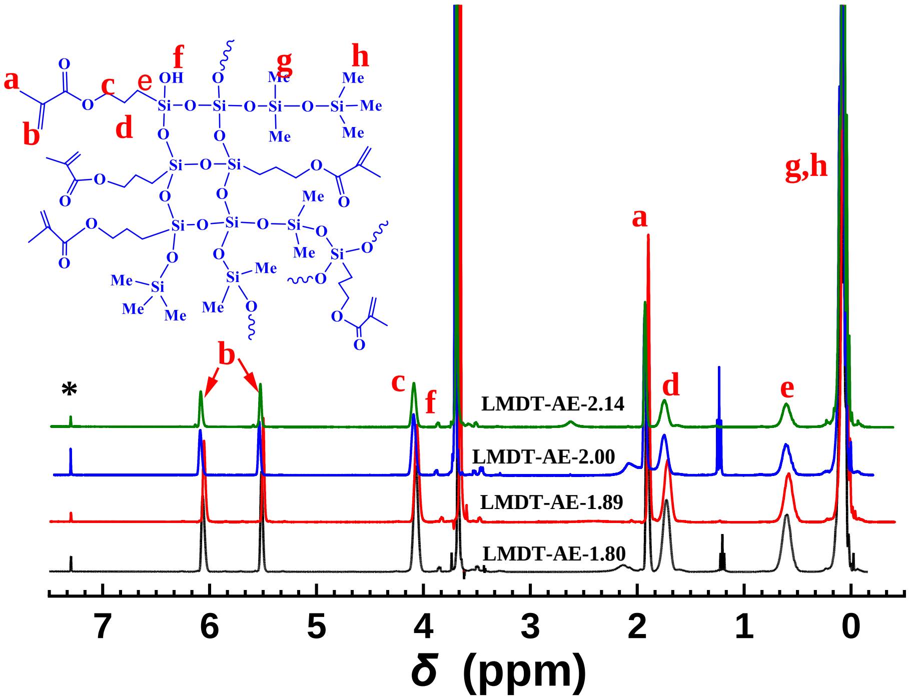


Fig. S1. $^{1}$H NMR spectrum of various LMDT−AE ("*" denotes the proton signals of chloroform, $\delta_{iso}$= 7.26 ppm)

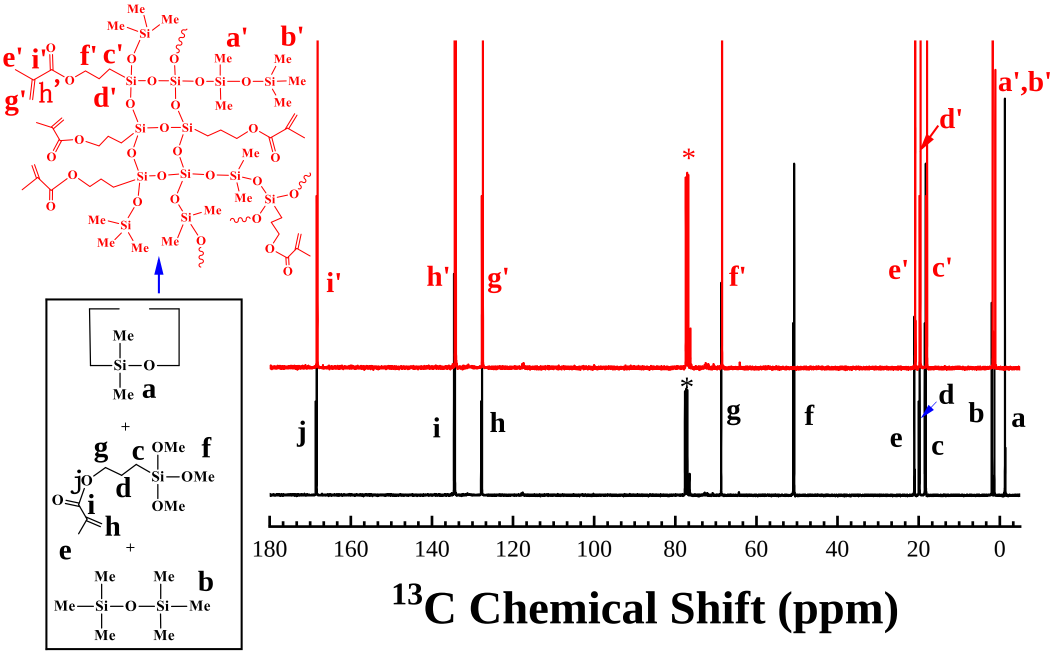


Fig. S2. $^{13}$C NMR spectrum of LMDT−AE before and after reaction ("*" denotes the proton signals of chloroform, $\delta_{iso}$ = 77.36 ppm)

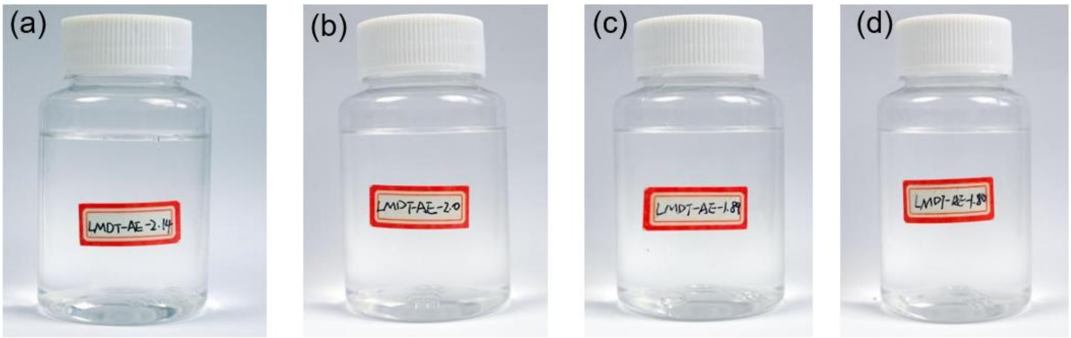


Fig. S3. Appearance of various LMDT−AE. (a: LMDT−AE−2.14; b: LMDT−AE−2.00; c: LMDT−AE−1.89, d: LMDT−AE−1.80)

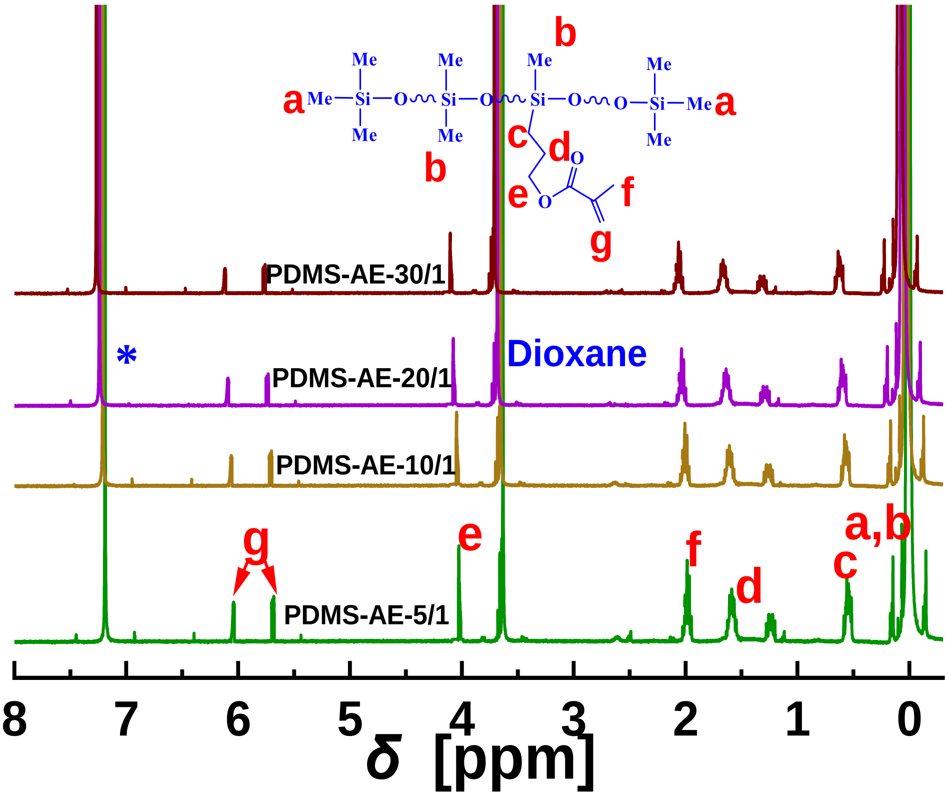


Fig. S4. $^{1}$H NMR spectrum of PDMS−AE ("*" denotes the proton signals of chloroform, $\delta_{iso}$= 7.26 ppm)

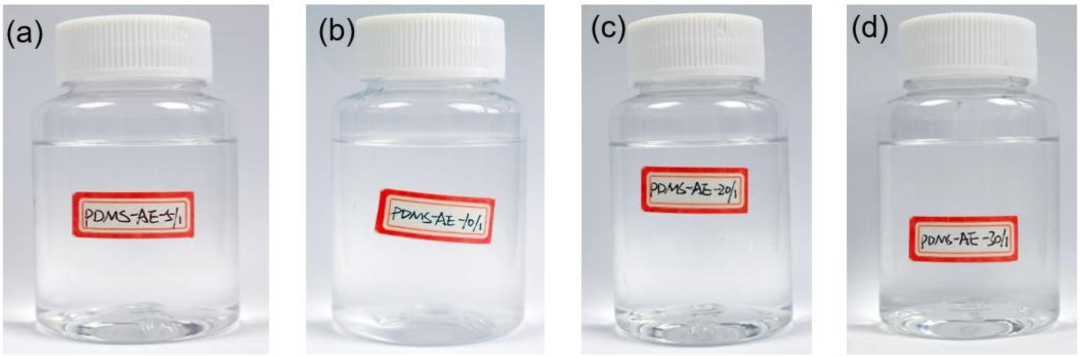


Fig. S5 Appearance of various PDMS−AE. (a: PDMS−AE−5/1; b: PDMS–AE−10/1; c: PDMS−AE−20/1, d: PDMS−AE−30/1)

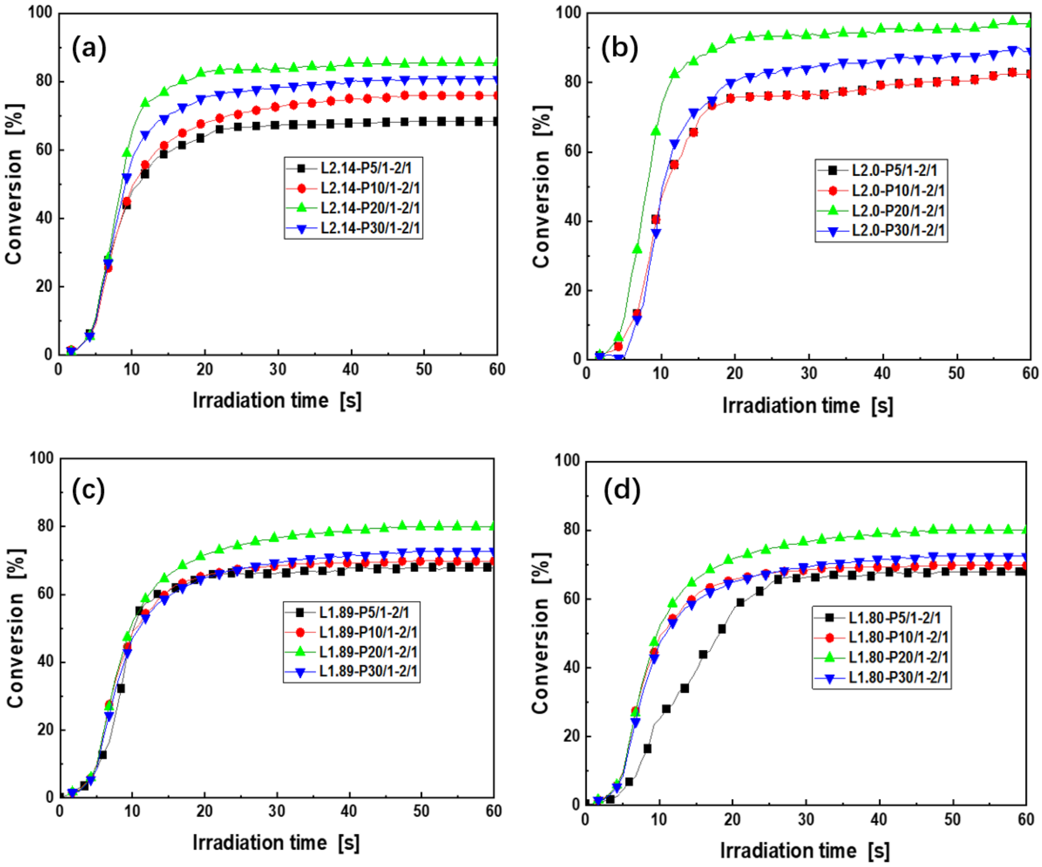


Fig. S6. Double bond conversion curve of 3D−AE was prepared from different PDMS−AE at the mass ratio of LMDT−AE to PDMS−AE was 2/1. (a)

LMDT−AE−2.14; (b) LMDT−AE−2.0; (c) LMDT−AE−1.89; (d) LMDT−AE−1.80

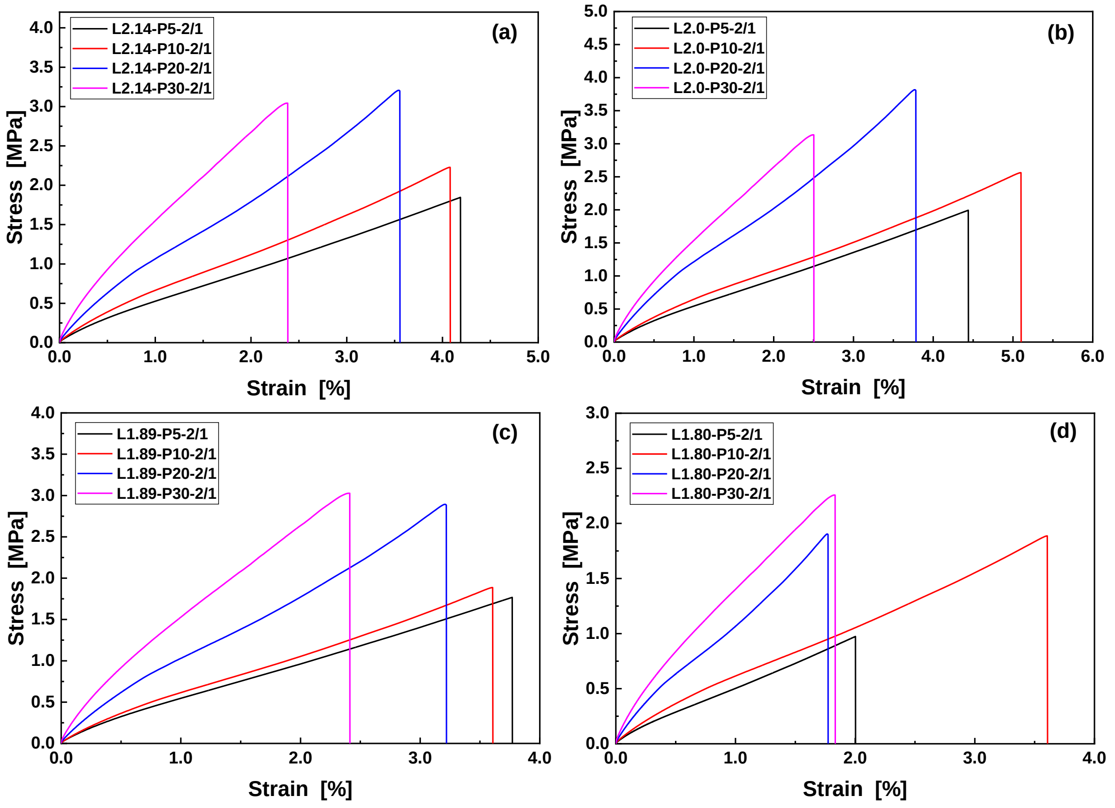


Fig. S7. The stress−strain curve of 3D−AE was prepared from different PDMS−AE when the mass ratio of LMDT−AE to PDMS−AE was 2/1, (a)LMDT−AE−2.14; (b) LMDT−AE−2.0; (c) LMDT−AE−1.89; (d) LMDT−AE−1.80

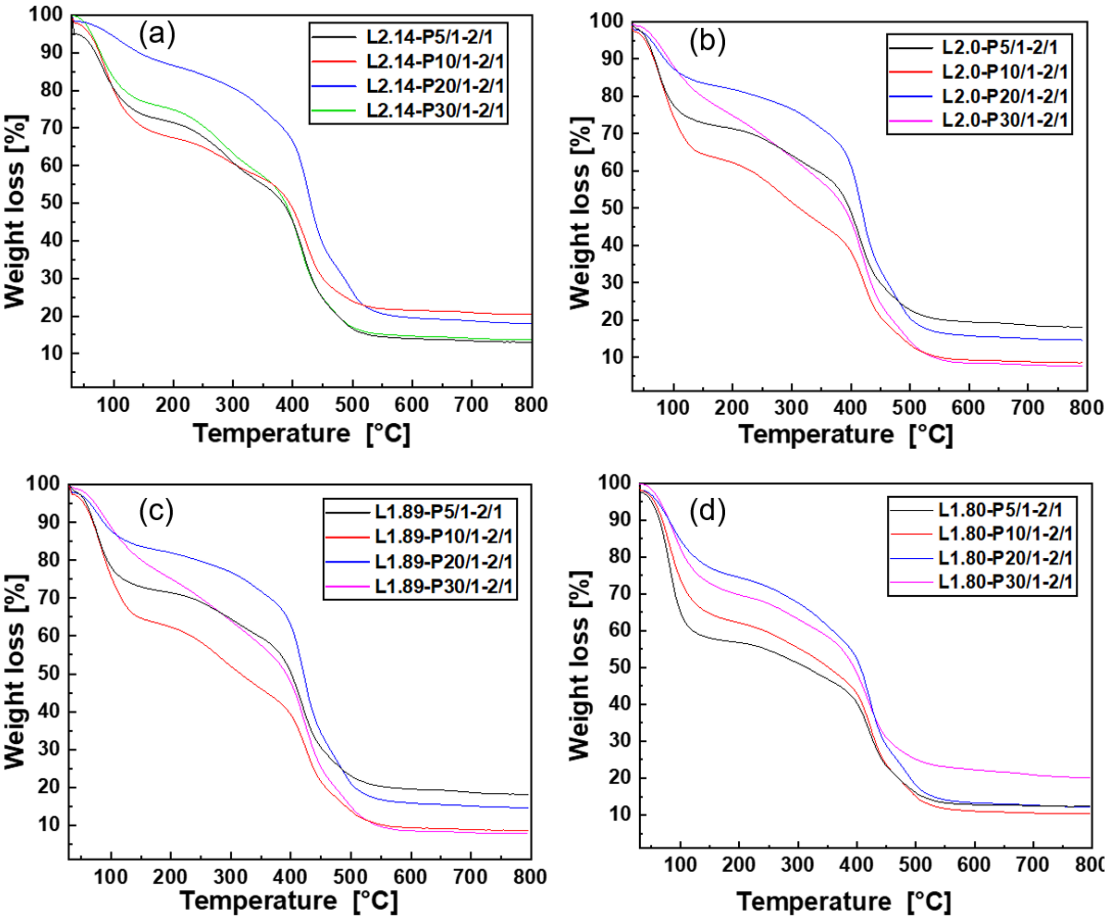


Fig. S8. The TGA curves of 3D−AE were prepared from different PDMS−AE when the mass ratio of LMDT−AE to PDMS−AE was 2/1. (a) LMDT−AE−2.14; (b) LMDT−AE−2.0; (c) LMDT−AE−1.89; (d) LMDT−AE−1.80

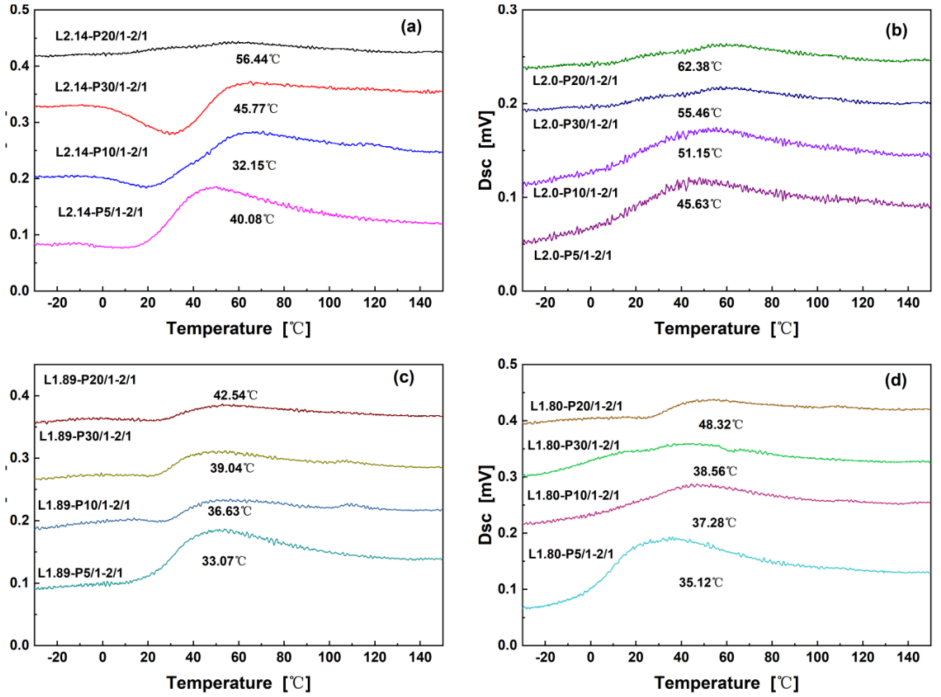


Fig. S9 When the mass ratio of LMDT−AE to PDMS−AE was 2/1, the DSC curve of 3D−AE was prepared from different PDMS−AE. (a) LMDT−AE−2.14; (b) LMDT−AE−2.0; (c) LMDT−AE−1.89; (d) LMDT−AE−1.80 (The experimental uncertainty is ±0.1 °C)

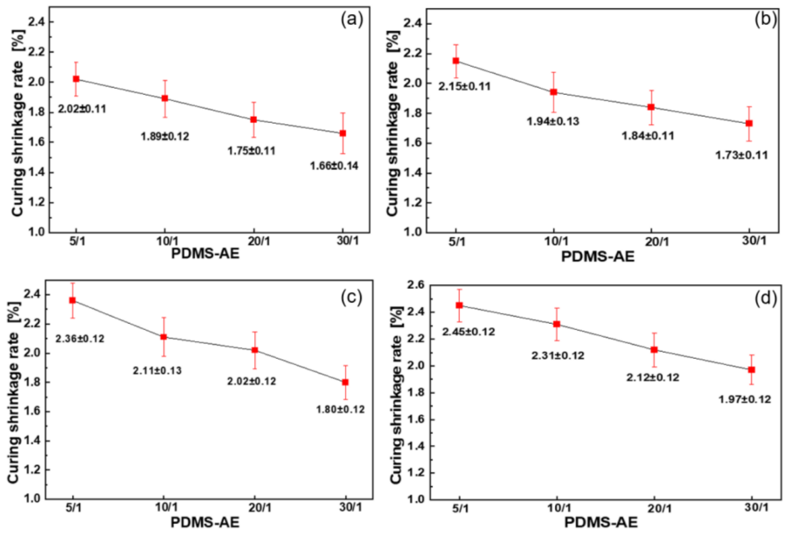


Fig. S10 When the mass ratio of LMDT−AE to PDMS−AE was 2/1, curing shrinkage rate of 3D−AE was prepared from different PDMS−AE. (a)LMDT−AE−2.14; (b) LMDT−AE−2.0; (c) LMDT−AE−1.89; (d) LMDT−AE−1.80

Table S1. Formulas for 3D−printing silicone resin composition 3D−AE

| **Sample** | MDT−AE | | PDMS−AE | | TPO−L [a] |
|---|---|---|---|---|---|
| | Type | Amount | Type | Amount | |
| L2.14−P5/1−2/1 | LMDT−AE−2.14 | 200g | PDMS−AE−5/1 | 100g | 3g |
| L2.14−P10/1−2/1 | | | PDMS−AE−10/1 | 100g | 3g |
| L2.14−P20/1−2/1 | | | PDMS−AE−20/1 | 100g | 3g |
| L2.14−P30/1−2/1 | | | PDMS−AE−30/1 | 100g | 3g |
| L2.0−P5/1−2/1 | LMDT−AE−2.00 | 200g | PDMS−AE−5/1 | 100g | 3g |
| L2.0−P10/1−2/1 | | | PDMS−AE−10/1 | 100g | 3g |
| L2.0−P20/1−2/1 | | | PDMS−AE−20/1 | 100g | 3g |
| L2.0−P30/1−2/1 | | | PDMS−AE−30/1 | 100g | 3g |
| L1.89−P5/1−2/1 | LMDT−AE−1.89 | 200g | PDMS−AE−5/1 | 100g | 3g |
| L1.89−P10/1−2/1 | | | PDMS−AE−10/1 | 100g | 3g |
| L1.89−P20/1−2/1 | | | PDMS−AE−20/1 | 100g | 3g |
| L1.89−P30/1−2/1 | | | PDMS−AE−30/1 | 100g | 3g |
| L1.80−P5/1−2/1 | LMDT−AE−1.80 | 200g | PDMS−AE−5/1 | 100g | 3g |
| L1.80−P10/1−2/1 | | | PDMS−AE−10/1 | 100g | 3g |
| L1.80−P20/1−2/1 | | | PDMS−AE−20/1 | 100g | 3g |
| L1.80−P30/1−2/1 | | | PDMS−AE−30/1 | 100g | 3g |
| L2.0−P20/1−3/1 | LMDT−AE−2.00 | 300g | PDMS−AE−20/1 | 100g | 4g |
| L2.0−P20/1−1/1 | | 100g | | 100g | 2g |
| L2.0−P20/1−1/2 | | 50g | | 100g | 1.5g |

[a] Amount of TPO−L: 1.00 wt.%

Table S2. AE contents of LMDT−AE

| AE contents[1] (mol/100g) | LMDT−AE−2.14 | LMDT−AE−2.00 | LMDT−AE−1.89 | LMDT−AE−1.80 |
|---|---|---|---|---|
| Theoretical value | 0.1570 | 0.1841 | 0.2826 | 0.3348 |
| Test value | 0.1532 | 0.1792 | 0.2743 | 0.3250 |
| Error | 2.42 % | 2.67 % | 2.92 % | 2.95 % |

[1] The analytical balance is accurate to 0.0001g, hence the measured AE content should be retained to four significant figures.

Table S3. GPC data of LMDT−AE

| Sample | GPC | | |
|---|---|---|---|
| | $M_n$ (g/mol) | $M_w$ (g/mol) | *PDI* |
| LMDT−AE−2.14 | 5803 | 6004 | 1.03 |
| LMDT−AE−2.00 | 2304 | 3817 | 1.66 |
| LMDT−AE−1.89 | 2605 | 4796 | 1.84 |
| LMDT−AE−1.80 | 4110 | 6130 | 1.49 |

Table S4. Physical properties of LMDT−AE

| Sample | Appearance | Viscosity (mPa·s) | Density (g/cm$^3$) | Refractive index (25 °C) | Transmittance 450 nm (%) |
|---|---|---|---|---|---|
| LMDT−AE−2.14 | Colorless and transparent dope | 520±15 | 1.0768±0.0135 | 1.4382±0.0012 | 95.28 |
| LMDT−AE−2.00 | | 315±9 | 1.0368±0.0128 | 1.4263±0.0015 | 87.78 |
| LMDT−AE−1.89 | | 423±12 | 1.0644±0.0141 | 1.4277±0.0013 | 90.31 |
| LMDT−AE−1.80 | | 550±18 | 1.0809±0.0150 | 1.4295±0.0010 | 89.22 |

Table S5. AE contents of PDMS−AE

| AE contents[1] (mol/100g) | PDMS−AE−5/1 | PDMS−AE−10/1 | PDMS−AE−20/1 | PDMS−AE−30/1 |
|---|---|---|---|---|
| Theoretical value | 0.1799 | 0.1080 | 0.0600 | 0.0416 |
| Test value | 0.1713 | 0.1032 | 0.0576 | 0.0401 |
| Error | 4.78% | 4.44% | 4.00% | 3.61% |

[1] The analytical balance is accurate to 0.0001g, hence the measured AE content should be retained to four significant figures.

Table S6. GPC data of PDMS−AE

| Sample | GPC | | |
|---|---|---|---|
| | $M_n$ (g/mol) | $M_w$ (g/mol) | *PDI* |
| PDMS−AE−5/1 | 1024 | 1090 | 1.16 |
| PDMS−AE −10/1 | 1066 | 1141 | 1.12 |
| PDMS−AE −20/1 | 1311 | 1716 | 1.08 |
| PDMS−AE −30/1 | 1338 | 1748 | 1.03 |

Table S7. Physical properties of PDMS−AE

| Sample | Appearance | Viscosity (MPa·s) | Density (g/cm$^3$) | Refractive index (25 °C) | Transmittance 450 nm (%) |
|---|---|---|---|---|---|
| PDMS−AE−5/1 | Colorless and transparent dope | 292±15 | 1.0472±0.0170 | 1.4295±0.0011 | 94.56 |
| PDMS−AE−10/1 | | 330±13 | 0.9614±0.0213 | 1.4256±0.0014 | 97.00 |
| PDMS−AE−20/1 | | 388±14 | 0.9603±0.0255 | 1.4237±0.0012 | 97.45 |
| PDMS−AE−30/1 | | 404±10 | 0.9573±0.0193 | 1.4198±0.0017 | 94.56 |

Table S8. TGA date of various 3D−AE

| Sample | $T_{5\%}$ (°C)[a] | $T_{50\%}$ (°C)[b] | $T_{max}$ (°C)[c] | Residual rate (%)[d] |
|---|---|---|---|---|
| L2.14−P30/1−2/1 | 59.86 | 388.86 | 358.36 | 13.76 |
| L2.14−P20/1−2/1 | 93.26 | 428.53 | 376.03 | 18.06 |
| L2.14−P10/1−2/1 | 58.36 | 393.16 | 317.66 | 20.61 |
| L2.14−P5/1−2/1 | 38.79 | 383.89 | 314.79 | 13.09 |
| L2.00−P30/1−2/1 | 69.37 | 388.87 | 314.37 | 7.75 |
| L2.00−P20/1−2/1 | 78.26 | 401.53 | 420.24 | 12.23 |
| L2.00−P10/1−2/1 | 53.98 | 313.12 | 321.00 | 8.67 |
| L2.00−P5/1−2/1 | 59.88 | 398.08 | 316.08 | 18.15 |
| L1.89−P30/1−2/1 | 67.29 | 388.56 | 317.46 | 7.75 |
| L1.89−P20/1−2/1 | 63.31 | 418.57 | 418.32 | 14.64 |
| L1.89−P10/1−2/1 | 53.59 | 313.38 | 321.77 | 8.67 |
| L1.89−P5/1−2/1 | 54.49 | 398.97 | 320.01 | 18.15 |
| L1.80−P30/1−2/1 | 67.53 | 395.52 | 372.24 | 20.11 |
| L1.80−P20/1−2/1 | 58.26 | 401.53 | 420.24 | 12.28 |
| L1.80−P10/1−2/1 | 55.36 | 348.52 | 345.34 | 10.31 |
| L1.80−P5/1−2/1 | 52.63 | 308.41 | 315.24 | 12.37 |
| L2.0−P20/1−3/1 | 66.33 | 390.12 | 368.53 | 21.29 |
| L2.0−P20/1−1/1 | 55.36 | 348.52 | 345.34 | 10.31 |
| L2.0−P20/1−1/2 | 52.63 | 308.41 | 315.24 | 12.37 |

[a] $T_{5\%}$: Temperature corresponding to 5 wt % weight loss

[b] $T_{50\%}$: Temperature corresponding to 50 wt % weight loss

[c] $T_{max}$: emperature corresponding to maximum weight loss rate

[d] Residual rate: The residual ratio of thermal decomposition corresponding to 800 °C

Table S9. Error between the design value of cylinder diameter and the actual measurement value

| Sample | Design dimensions | Measure dimensions | Error value |
|---|---|---|---|
| 1 | 0.70 mm | 0.728 mm | 0.028 mm |
| 2 | 0.95 mm | 0.978 mm | 0.028 mm |
| 3 | 1.00 mm | 1.030 mm | 0.030 mm |
| 4 | 1.35 mm | 1.360 mm | 0.010 mm |
| 5 | 1.80 mm | 1.820 mm | 0.020 mm |
| 6 | 2.20 mm | 2.210 mm | 0.010 mm |
